# Reframing quercetin as a promiscuous inhibitor against SARS-CoV-2 main protease

**DOI:** 10.1073/pnas.2309289120

**Published:** 2023-09-05

**Authors:** Haohao Yan, Rui Zhang, Xiaoping Liu, Yanchang Wang, Yunyu Chen

**Affiliations:** ^a^Institute for Drug Screening and Evaluation, Wannan Medical College, Wuhu 241002, China; ^b^Department of Biomedical Sciences, College of Medicine, Florida State University, Tallahassee, FL 32306

Traditional Chinese medicine has made contributions to the treatment of coronavirus disease 2019 (COVID-19) because of its favorable efficacy, such as Huashi Baidu decoction (Q-14) (1, 2). Recently, quercetin, a main component of Q-14, has been identified as a potent inhibitor against severe acute respiratory syndrome coronavirus 2 (SARS-CoV-2) main protease (Mpro) using an integrative pharmacological strategy, and its inhibitory effect on Mpro is examined by the fluorescence resonance energy transfer (FRET) assay with the half-maximal inhibitory concentration (IC_50_) value of 22.47 μM (3). Considering the potential of quercetin in COVID-19 treatment, a rigorous validation for its Mpro inhibition is necessary.

**Fig. 1.**
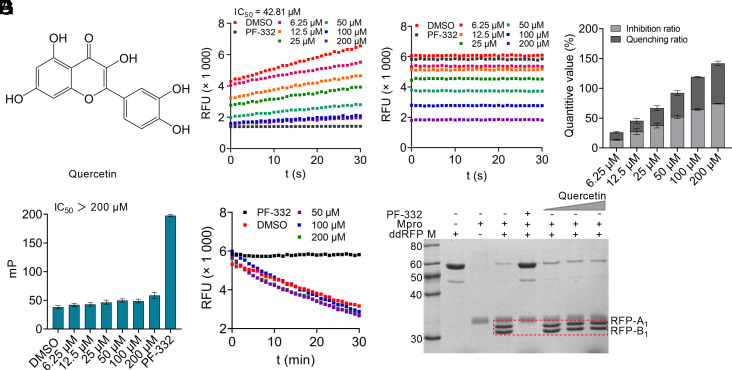
Inhibitory effect of quercetin on SARS-CoV-2 Mpro in vitro. (*A*) The chemical structure of quercetin. (*B*) Inhibition of Mpro by quercetin using FRET assay. (*C*) The fluorescence quenching effect of quercetin on MCA-AVLQ fragment in the FRET assay. MCA: 7-methoxycoumarin-4-acetic acid. (*D*) The comparison between Mpro inhibition and fluorescence quenching effect of quercetin in the FRET assay. (*E*) Inhibition of Mpro by quercetin using FP assay. The FRET and FP assays were carried out as previously described (4, 5, 7). The IC_50_ value of quercetin was shown. Nirmatrelvir (PF-332, 1 μM) and DMSO served as the positive and negative controls, respectively. (*F* and *G*) Inhibition of Mpro by quercetin using ddRFP assay. The time course trajectories of ddRFP biosensor in the presence of quercetin at the indicated concentrations were recorded every minute for 30 min by a microplate reader (BioTek). A fluorescent ddRFP biosensor produces a high RFU value in the presence of Mpro inhibitors, whereas the cleavage by Mpro generates two separate RFP fragments with a low RFU signal. In the gel-based assay, the ddRFP biosensor (55 kDa) can be cleaved by Mpro (34 kDa) to generate RFP-A_1_ (top band, 29 kDa) and RFP-B_1_ fragments (bottom band, 26 kDa). The testing concentration of quercetin was 50, 100, or 200 μM. Nirmatrelvir (PF-332, 10 μM) and DMSO were used as the positive and negative controls, respectively. The ddRFP assay was performed based on our previous publications (6, 7).

We have developed a systematic high-throughput screening (HTS) platform for the discovery and assessment of Mpro inhibitors, including FRET, fluorescence polarization (FP), and dimerization-dependent red fluorescent protein (ddRFP) assays (4–7). With these assays, we previously demonstrated that baicalein is a nonspecific Mpro inhibitor (7). Herein, we rigorously evaluated the inhibition of Mpro by quercetin in vitro using these HTS assays (Fig. 1*A*). To ensure the reliability of these assays, nirmatrelvir (PF-07321332, PF-332) served as a positive control in the presence of dithiothreitol (DTT) (7). Using FRET assay, our results showed that quercetin exhibits apparent inhibition against Mpro (IC_50_ = 42.81 μM) (Fig. 1*B*). However, the presence of quercetin at the testing concentrations was able to quench the fluorescence signal of MCA-AVLQ fragment, which is generated by the cleaved FRET substrate (Fig. 1*C*). Importantly, this quenching effect fully contributed to the observed Mpro inhibition by quercetin in the FRET assay, suggesting that this inhibition is false positive (Fig. 1*D*). We speculate that the overlap region between the absorption spectrum of quercetin and an emission wavelength of MCA-AVLQ fragment may cause this fluorescence quenching effect (8).

We further evaluated Mpro inhibition by quercetin using FP assay. As expected, quercetin did not show any inhibition against Mpro using this assay (IC_50_ > 200 μM) (Fig. 1*E*). Moreover, a versatile ddRFP biosensor has been used to assess Mpro inhibitors by monitoring the change of relative fluorescence unit (RFU) value as well as the cleavage of ddRFP biosensor using SDS-PAGE analysis (6). Intriguingly, a low RFU value was recorded over time with a concentration of quercetin at 200 μM, indicating that quercetin could not inhibit the cleavage of ddRFP biosensor catalyzed by Mpro (Fig. 1*F*). Consistently, quercetin did not show obvious inhibition against Mpro using SDS-PAGE assay because we detected efficient cleavages of ddRFP biosensor even in the presence of quercetin (Fig. 1*G*). Therefore, our results suggest that the in vitro inhibition of Mpro by quercetin is false positive, and the fluorescence quenching effect caused by natural products should be considered when evaluating Mpro inhibitors.

In conclusion, our data indicate that quercetin is a promiscuous Mpro inhibitor based on the results using a set of in vitro assays. These results suggest a stringent in vitro validation with diverse biochemical assays is essential for the discovery of Mpro inhibitors in the future.
